# Supplementary material for: Mechanistic Analysis of CCP1 in Generating ΔC2 α-Tubulin in Mammalian Cells and Photoreceptor Neurons
Source: Biomolecules. 2023 Feb 12;13(2):357. doi: 10.3390/biom13020357 (PMC9952995; doi:10.3390/biom13020357)
Supplement: Supplementary file 1 [file biomolecules-13-00357-s001.zip › biomolecules-2153786-supplementary.pdf]

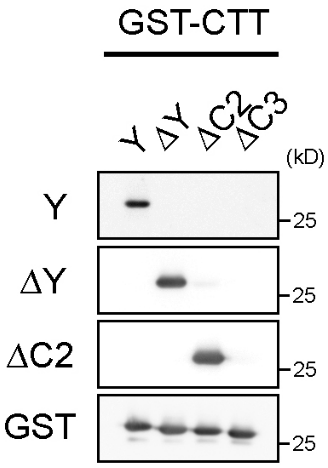

Figure S1. Specificity of α-CTT antibodies used in this study.
Purified GST-CTT proteins (Y, ΔY, ΔC2 or ΔC3 forms) were blotted with rat monoclonal antibody against Y-α-tubulin (YL1/2), recombinant antibodies against ΔY-α-tubulin (RM444) or ΔC2-α-tubulin (RM447) and anti-GST antibodies. All α-CTT antibodies are specific to corresponding forms of GST-CTT proteins.

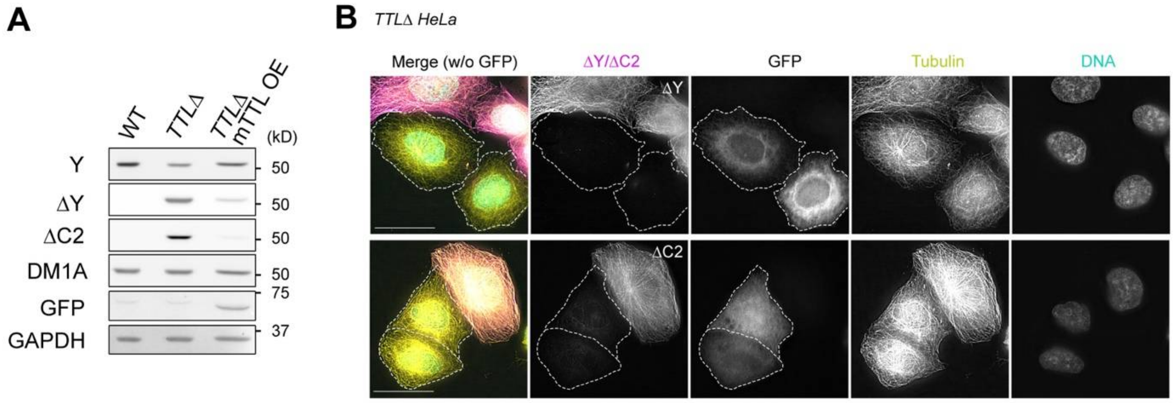

Figure S2. The levels of ΔY- and ΔC2-α-tubulin decrease in *TTLΔ* cells transiently expressing GFP-mTTL.

EGFP-tagged mouse TTL was overexpressed in *TTLΔ* cells and the levels of ΔY- and ΔC2-α-tubulin were analyzed by immunoblotting (A) and immunofluorescence (B). Dotted lines indicate cells expressing EGFP-mTTL. Scale bars, 25 μm.

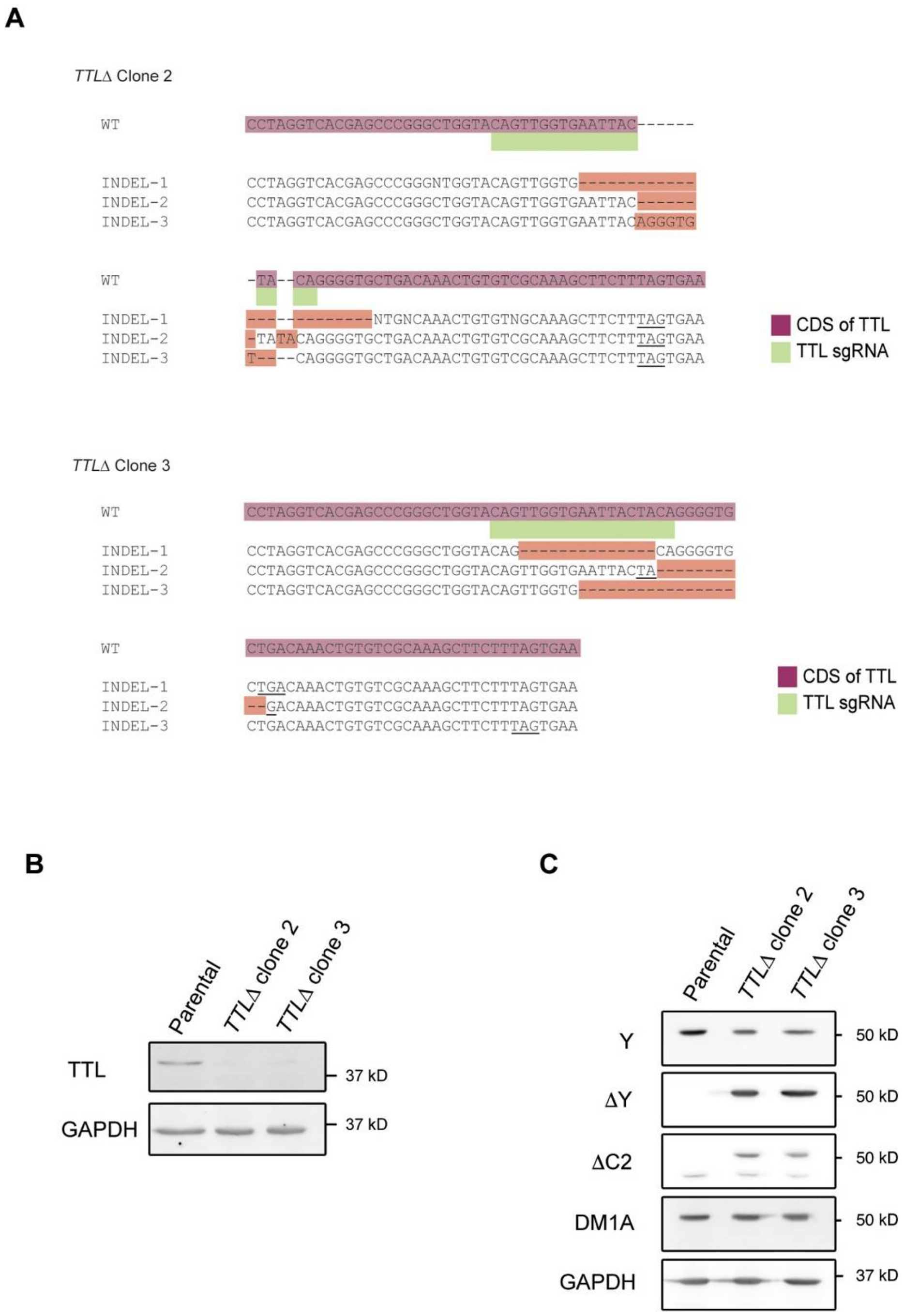

Figure S3. Characterization of additional *TTL*Δ clones.
(**A**) Detection of CRISPR/Cas9-induced INDELs in *TTL*Δ clones 2 and 3. Three independent INDELs, all of which resulted in early stop codons (underlined) were identified for each clone. (**B** and **C**) Immunoblotting of whole cell lysates prepared from the parental HeLa cell line and *TTL*Δ clones 2 and 3. TTL was at nearly undetectable levels (B) and concomitantly the ΔC2-tubulin level increased (C).

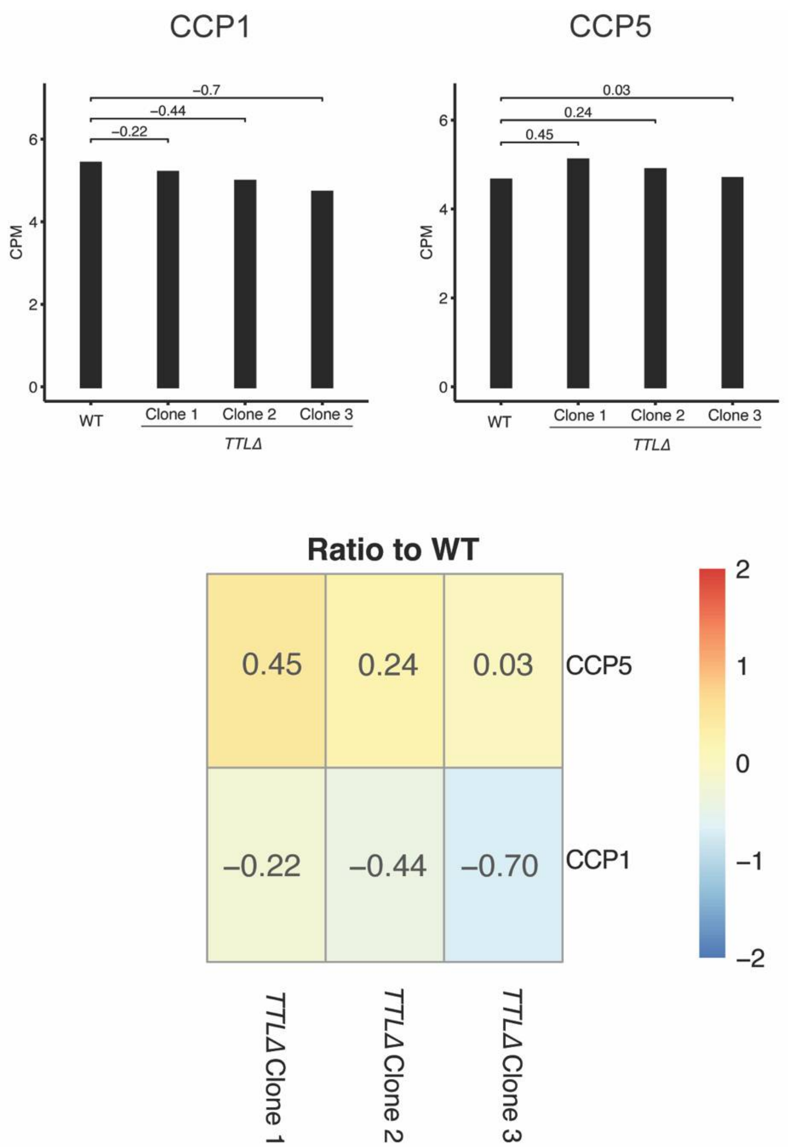

Figure S4. CCP1 and CCP5 are the only expressed cytosolic carboxypeptidases in the parental HeLa and *TTL*Δ lines.

Quantifications of transcript levels of CCP1 and CCP5 in the parental HeLa and *TTL*Δ lines.

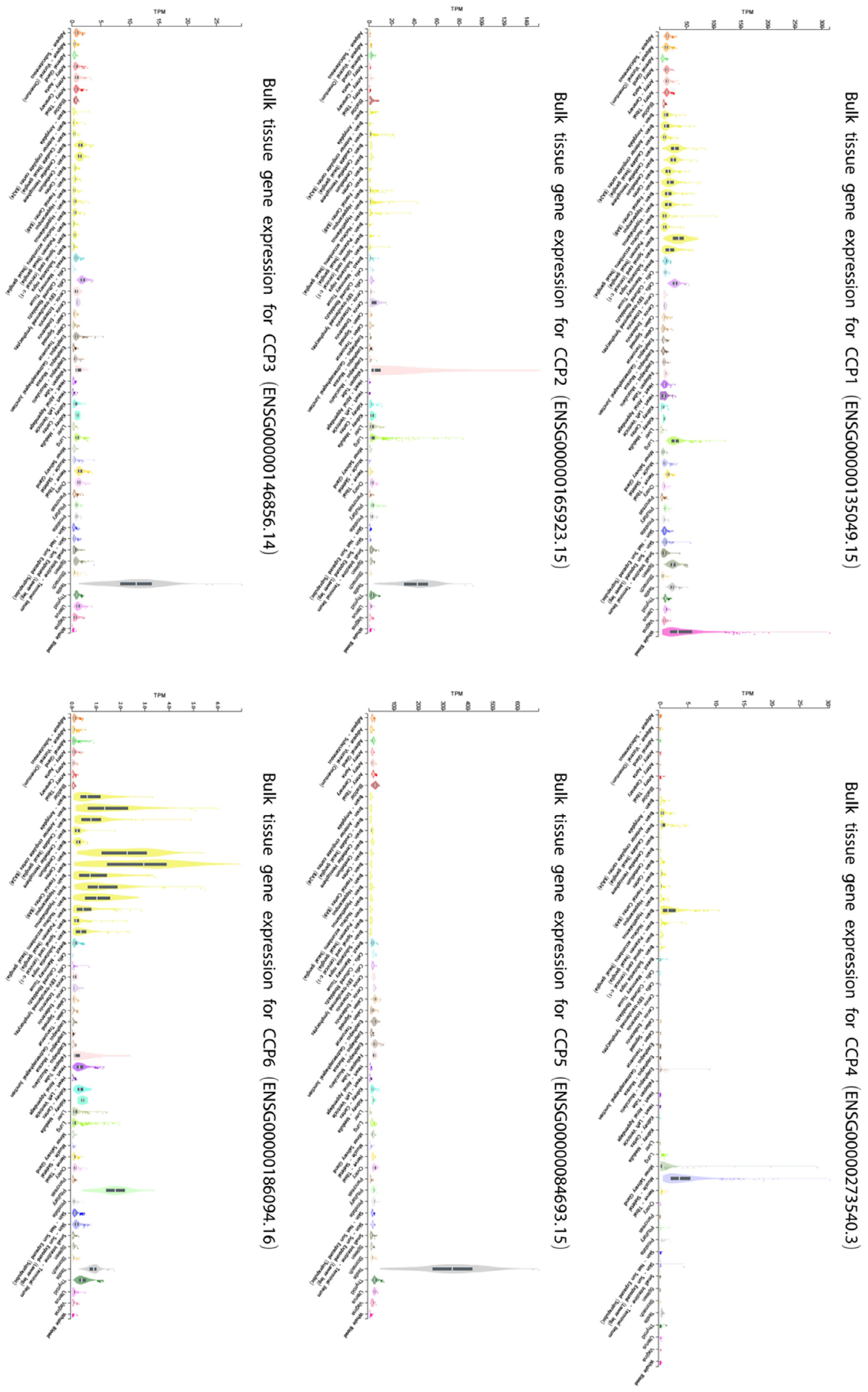

Figure S5. Expression levels of CCP genes in various tissues.
Plots were generated from the GTEx Portal Release V8 at gtexportal.org on 11/22/22. The NIH funded GTEx project, developed and hosted by the Broad Institute, contains RNAseq data generated from a collection of tissues. They used the STAR v2.5.3a aligner with the human reference genome GRCh38 and GENECODE v26 annotation. For each tissue source, TPM (Transcripts Per Million) are plotted as violin plots and box plots containg the, median, 25th and 75th percentile. Samples outside 1.5 times interquartile range are shown as individual dots.

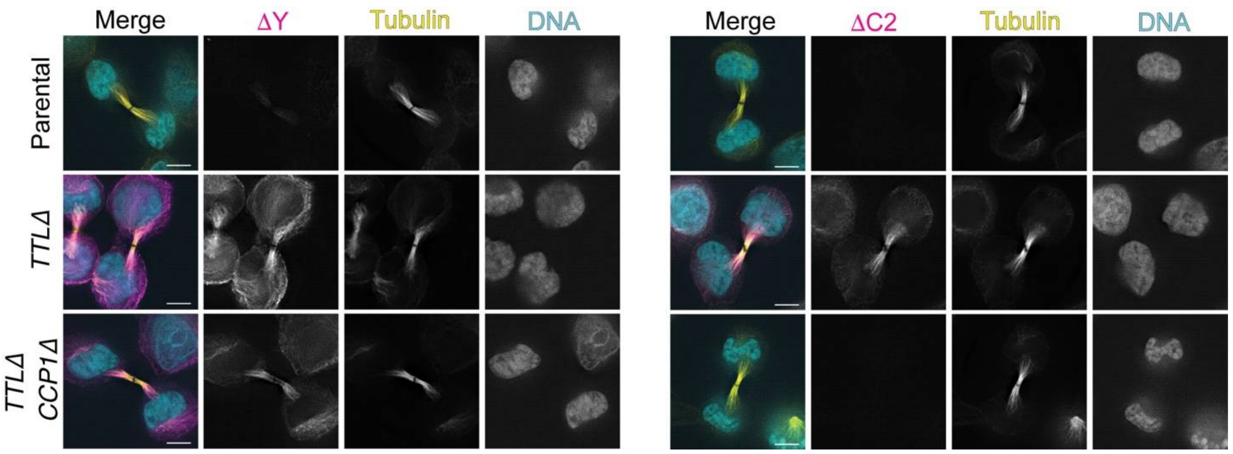

Figure S6. Immunofluorescence staining of *TTL*Δ *CCP1*Δ cells undergoing cytokinesis.
In merged images, ΔY- or ΔC2-α-tubulin is shown in magenta, total α-tubulin (DM1A staining) in yellow and DNA (DAPI staining) in cyan. Scale bars, 5 µm. Related to Figure 3.

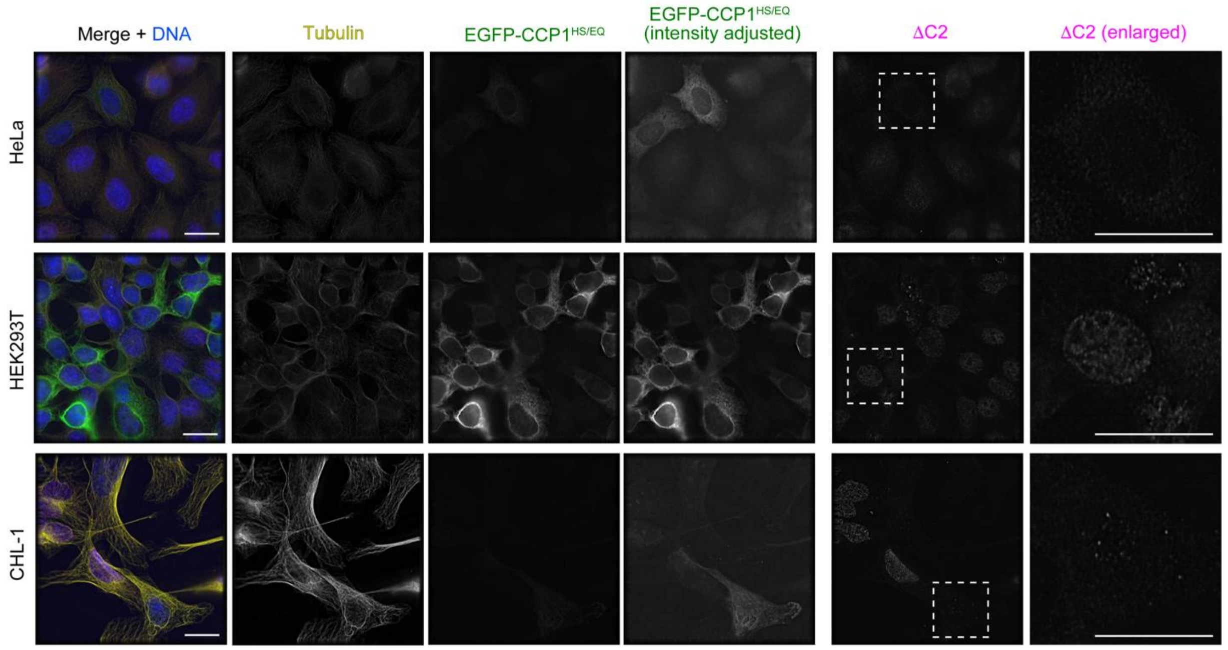

Figure S7. Transient expression of CCP1 mutant in HeLa, HK293T and CHL-1 cells. Immunofluorescence images of cells transiently expressing a catalytically inactive version of EGFP-CCP1 mutant (HS/EQ) are shown. Total α-tubulin (DM1A staining) is shown in yellow, EGFP-CCP1 $^{HS/EQ}$ in green, ΔC2-α-tubulin in magenta and DNA (DAPI staining) in blue. Scale bars, 20 μm. Related to Figure 4.

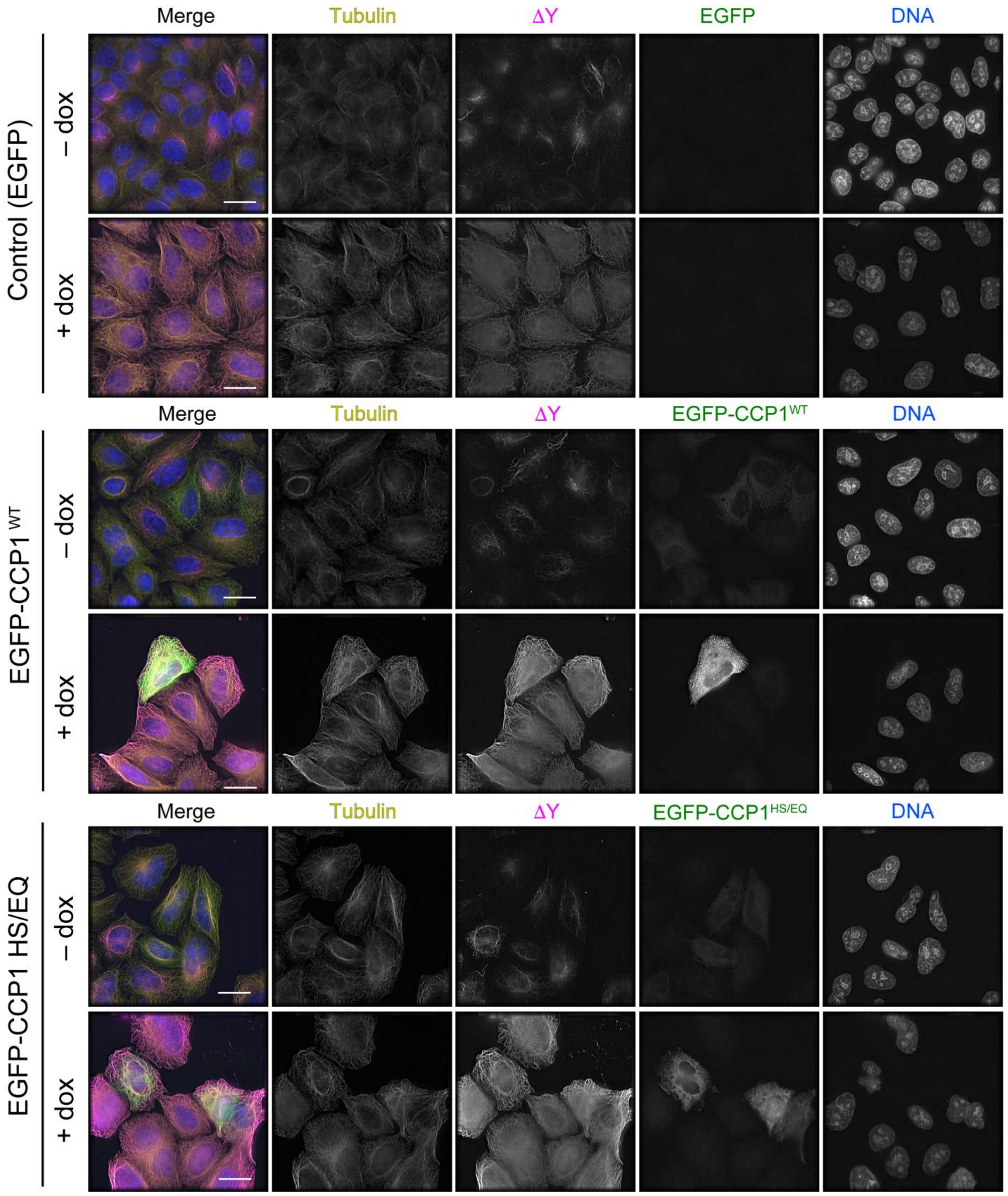

Figure S8. Immunofluorescence images of ΔY-α-tubulin in HeLa cells expressing VASH1-SVBP. In merged images, total α-tubulin (DM1A staining) is shown in yellow, ΔY-α-tubulin in magenta, EGFP in green, and DNA (DAPI staining) in blue. Scale bars, 20 μm. Related to Figure 5.

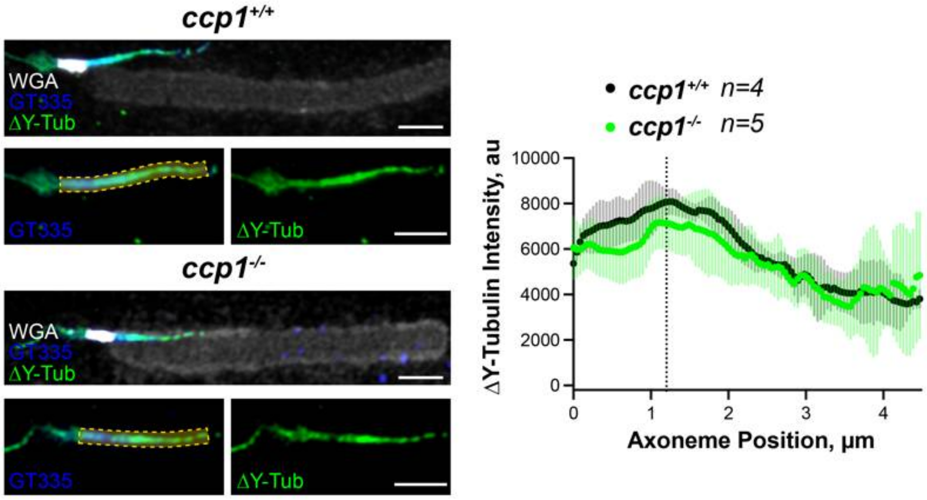

Figure S9. ΔY-α-tubulin staining is not changed in the photoreceptor axoneme of *ccp1*$^{-/-}$ mouse retinas

Representative Airyscan images of isolated outer segments from P15 *ccp1*$^{+/+}$ and *ccp1*$^{-/-}$ retinas stained with anti-GT335 polyglutamylation (blue), WGA (gray), and anti-ΔY-α-tubulin (green) antibodies. Scale bar, 2 µm. Averaged intensity plots shown to right.

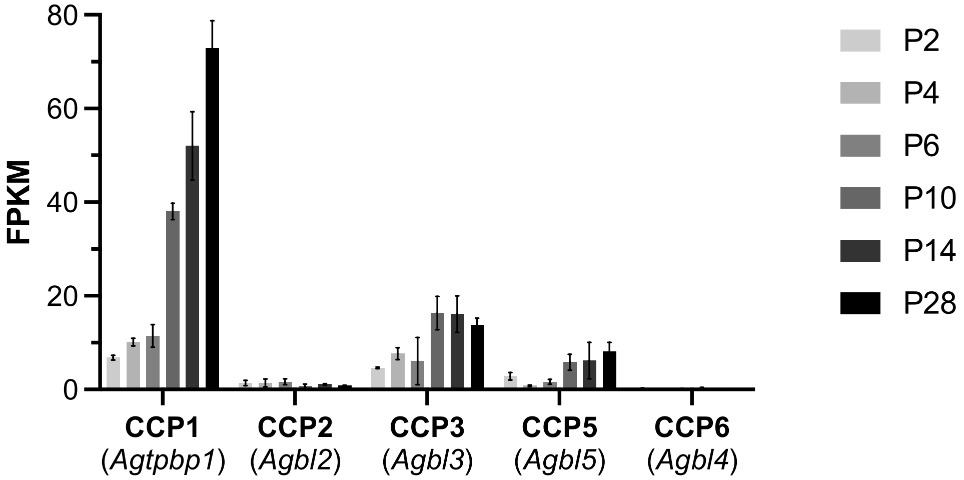

Figure S10. Expression levels of CCPs in photoreceptor neurons at the indicated days of post-natal development.

Name	Sequence (5’–3’)
oMG68	CTGCGTTCGTCAAGCTGTGACAAGCTTCGAATTCTGCAGTCG
oMG69	TCGTCGCGCACCACGAAGGTTCTGAGTCCGGACTTGTACAGCTCG
oMG70	TGTACAAGTCCGGACTCAGAACCTTCGTGGTGCGC
oMG71	ACTGCAGAATTCGAAGCTTGTCACAGCTTGACGAACGCAGC
TH511	ACCCTCTCCTTCAACCGATGAATTAAGCTTGAGCTC
TH514	GAGGAAGAAGGAGAGTGACTGACTGACGATCTGCCT
TH515	GAGGAAGAAGGATGACTGACTGACGATCTGCCT
TH738	GATCTCGAGCTCAAGCTTCGAGCAAGTTAAAAGTGATACC
TH739	CGCGGTACCGTCGACTGCAGTCAAGGTAGGTATGTTCTTG
TH744	CTTGCATTAGTTTGTCCAGGACTTACCCGAGCAG
TH812	GATCTCGAGCTCAAGCTTCGATGTTCCCAGCTTTGGAAAC
TH813	CGCGGTACCGTCGACTGCAGCTACGGGTATGTGTATATGTG
TH817	GATCTCGAGCTCAAGCTTCGATGTCAGAAGATTCAGAAAAG
TH818	CGCGGTACCGTCGACTGCAGTCATCTCAATAAAAATGTCAGTTC
TH888	GATCTCGAGCTCAAGCTTCGATGGCCGAACAAGAAGCTAG
TH889	TCAGATGTGTCTGCAGAAGCTTATCTATTACTTTG
TH890	GCTTCTGCAGACACATCTGAAGCGTGTC
TH891	CGCGGTACCGTCGACTGCAGTCAAGTGTCACAAACTGG
TH821	GATCTCGAGCTCAAGCTTCGATGGAGCTGCGCTGTGGG
TH822	CGCGGTACCGTCGACTGCAGTCATCCCTCTGCGAGTCG
TH895	GATCTCGAGCTCAAGCTTCGATGGCGGAGGGGAGCCAG
TH896	CGCGGTACCGTCGACTGCAGTTAAAAAGGGGTTGAAGGG

Table S1. Oligonucleotide DNA primers used in this study
